# Hepatic metastasis of mucinous adenocarcinoma with neuroendocrine differentiation harboring a rare HER2 R678Q mutation: a case report and literature review

**DOI:** 10.3389/fonc.2026.1814733

**Published:** 2026-06-18

**Authors:** Zhitao Chen, Chenchen Ding, Yangjun Gu, Qiyong Li

**Affiliations:** 1 Department of Hepatobiliary Surgery, Shulan (Hangzhou) Hospital Affiliated to Zhejiang Shuren University Shulan International Medical College, Hangzhou, China; 2 Affiliated Mental Health Centre and Hangzhou Seventh People’s Hospital, Zhejiang University School of Medicine, Hangzhou, Zhejiang, China

**Keywords:** cancer of unknown primary, HER2 mutation, mucinous adenocarcinoma, neuroendocrine differentiation, trastuzumab

## Abstract

**Background:**

Cancer of unknown primary (CUP) remains a diagnostic and therapeutic challenge, particularly when involving rare histological and molecular subtypes. Mucinous adenocarcinoma (MAC) with neuroendocrine differentiation is exceptionally rare, and its molecular characteristics are poorly understood.

**Materials and methods:**

We report a case of CUP initially presenting with multiple liver and bone metastases. Histopathological and immunohistochemical analyses of a liver biopsy confirmed MAC with neuroendocrine differentiation. Comprehensive molecular profiling was performed to identify potential actionable mutations.

**Results:**

Genomic analysis revealed a rare HER2 (ERBB2) R678Q mutation. The patient received FOLFOX chemotherapy with the addition of trastuzumab, based on HER2 status. However, the tumor demonstrated early resistance and continued progression despite subsequent treatment modification. The patient succumbed to disease progression within one year of diagnosis.

**Conclusion:**

This is the first reported case of MAC with neuroendocrine features harboring a HER2 R678Q mutation in the context of CUP. The case underscores the importance of integrating broad molecular testing into CUP diagnosis to uncover rare mutations with potential therapeutic implications. It also highlights the limited efficacy of current HER2-targeted therapies in rare mutations and the urgent need for more tailored treatment strategies.

## Introduction

Cancer of unknown primary (CUP) is characterized by histologically confirmed metastatic disease in which the primary tumor remains unidentified despite comprehensive diagnostic evaluation (1–3). CUP accounts for approximately 1–2% of all cancer diagnoses worldwide and represents a major clinical challenge due to its biological heterogeneity and generally poor prognosis (4, 5). Notably, nearly 70% of CUP cases are adenocarcinomas (6). Mucinous adenocarcinomas (MACs) are a rare and poorly understood subtype of malignancy, accounting for approximately 1% of all cancer diagnoses (7). Histologically, MACs are defined by abundant extracellular mucin comprising more than 50% of the tumor volume, within which malignant epithelial cells are arranged in clusters, layers, or as isolated cells embedded in a mucin-rich stroma (7, 8). MACs are known to arise from multiple potential primary sites, including the gastrointestinal tract, breast, pancreas, lung, and bladder, with metastases most frequently involving the ovaries, peritoneum, liver, and lungs (7, 9, 10). Despite advances in imaging, pathology, and molecular profiling, the diagnosis and optimal management of MACs presenting as CUP remain particularly challenging, underscoring the need for improved diagnostic strategies and individualized therapeutic approaches.

Human epidermal growth factor receptor 2 (HER2), encoded by *ERBB2*, is a transmembrane tyrosine kinase receptor involved in regulating cell growth, survival, and proliferation (11, 12). Its dysregulation drives oncogenesis by activating key signaling pathways, notably PI3K–AKT and MEK–ERK, leading to uncontrolled cellular proliferation and tumor progression (13, 14). Alterations in *ERBB2*, including gene amplification–driven overexpression and activating somatic mutations, are observed across a broad range of human malignancies (15, 16). HER2 overexpression has been documented in multiple epithelial cancers, most notably breast and gastric cancers, as well as colorectal, esophageal, bladder, cholangiocarcinoma, gallbladder, cervical, and endometrial cancers (17, 18). In addition to overexpression, *ERBB2* mutations are recurrent in several tumor types, with the highest frequencies reported in bladder cancer, followed by uterine and cervical cancers, colorectal cancer, lung cancer, and breast cancer (16, 19). HER2 mutations predominantly cluster in exon 20, with A775_G776insYVMA representing the most common insertion, followed by less frequent variants such as G776delinsVC and P780_Y781insGSP (20). Since the approval of trastuzumab in 1998 for HER2-positive breast cancer, HER2-targeted therapies have evolved considerably, encompassing monoclonal antibodies, tyrosine kinase inhibitors, and antibody-drug conjugates—broadening treatment options across various HER2-altered malignancies (21). However, data on HER2-mutant MACs of unknown primary origin remain extremely limited, and the biological behavior, therapeutic responsiveness, and optimal management strategies for this rare entity are still poorly understood.

We present a rare case of CUP that initially manifested with multiple liver metastases. Histopathological and immunohistochemical analysis revealed features consistent with an intestinal-type MAC, exhibiting neuroendocrine differentiation. Comprehensive genomic profiling identified a rare *ERBB2* (HER2) R678Q mutation, which, to our knowledge, represents the first documented case of a HER2 R678Q–mutant MAC of unknown primary origin with neuroendocrine differentiation. This case highlights a previously unrecognized molecular variant of CUP and expands the mutational spectrum of *ERBB2* in mucinous neoplasms. Moreover, it underscores the clinical importance of incorporating broad genomic testing in challenging CUP diagnoses to identify rare molecular alterations that may influence treatment strategies.

## Case report

A 50-year-old male patient presented in January 2025 with intermittent generalized seizures. The available clinical records revealed no remarkable past medical history, family history of malignancy, psychosocial history, or known hereditary genetic disorder. Abdominal magnetic resonance imaging (MRI) revealed multiple hypodense lesions in the liver. Tumor marker analysis showed a significantly elevated carcinoembryonic antigen (CEA) level of 93 ng/mL. Subsequent positron emission tomography–computed tomography (PET-CT) imaging demonstrated multiple liver metastases and widespread bone metastases, with increased fluorodeoxyglucose (FDG) uptake, raising suspicion for a gastrointestinal malignancy with liver and skeletal involvement. To further investigate the source, gastrointestinal endoscopy and biopsy were performed, which revealed low-grade dysplasia in colonic/rectal polyps and mucosal inflammation in the gastric antrum, but no definitive malignant tumors were identified. Unfortunately, the patient’s condition rapidly deteriorated due to spinal metastasis, and they developed generalized paralysis and incontinence, which prompted further clinical evaluation. The patient had not undergone any tumor-directed treatment before referral to our hospital.

In March 2025, the patient was referred to our hospital for specialized treatment. Laboratory results showed marked elevation of tumor markers, including alpha-fetoprotein (AFP) at 2.2 ng/mL, CEA at 124.7 ng/mL, carbohydrate antigen 19-9 (CA19-9) at 36.7 U/mL, ferritin at 696.5 ng/mL, cytokeratin 19 fragment (CYFRA21-1) at 24.8 ng/mL, and CA72–4 exceeding 600 IU/mL. Abdominal contrast-enhanced CT imaging revealed multiple hepatic lesions consistent with malignancy and vertebral metastases (**Figure 1**). After multidisciplinary discussion (MDT), it was concluded that the liver and bone metastases were not of primary origin, prompting further investigation into other potential primary sites of the malignancy. A guided ultrasound liver biopsy was performed, which revealed MAC with neuroendocrine differentiation (**Figures 2A–C**). Special staining showed negative results for D-PAS, PAS, and Masson trichrome, while reticulin, AB, and mucicarmine stains were positive. Immunohistochemistry results indicated the following: SATB2 (-), CDX2 (+), CD56 (-), CgA (-), Syn (+), CK7 (-), CK20 (+), KI67 70%, P53 (null mutation), and Her-2 (0). Based on the immunohistochemical profile, the tumor was most likely of gastrointestinal origin (**Figures 2D–I**).

**Figure 1:**
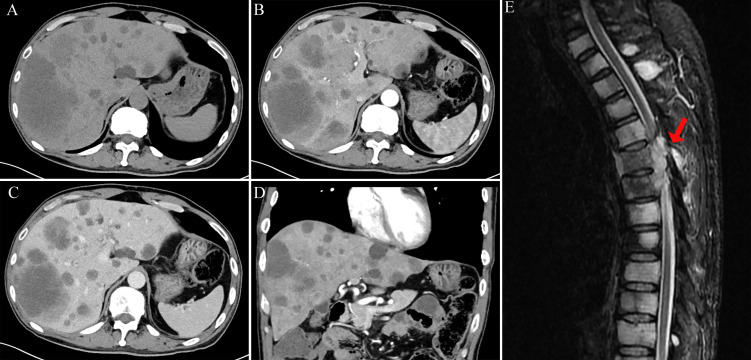
Baseline imaging studies obtained at the patient’s first presentation to our institution, including abdominal computed tomography (CT) and thoracolumbar magnetic resonance imaging (MRI). **(A)** Non-contrast CT scan revealing multiple hypoattenuating lesions scattered throughout the liver, highly suggestive of multifocal hepatic metastases. **(B)** Arterial-phase contrast-enhanced CT demonstrating the enhancement characteristics of the hepatic lesions. **(C)** Portal venous-phase contrast-enhanced CT further delineating lesion distribution and vascular characteristics. **(D)** Coronal reconstructed CT image illustrating the extent and anatomical distribution of the hepatic lesions. **(E)** Thoracic spine MRI showing metastatic involvement of the thoracic vertebrae with associated spinal cord compression.

**Figure 2:**
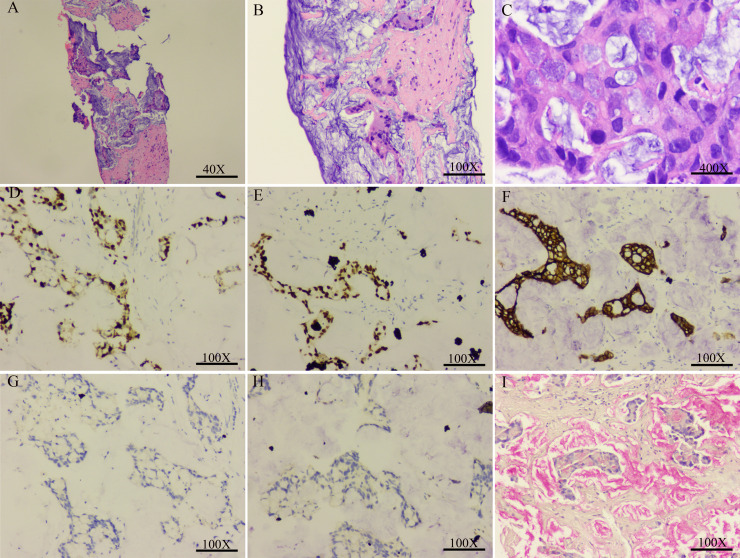
Histopathological and immunohistochemical findings of the ultrasound-guided liver biopsy specimen. **(A)** Hematoxylin and eosin (H&E) staining (×40) showing diffuse infiltration of tumor cells within the hepatic parenchyma accompanied by abundant extracellular mucin pools. **(B)** H&E staining (×100) demonstrating irregular glandular structures and clusters of malignant epithelial cells floating within mucin lakes. **(C)** H&E staining (×400) revealing marked cytologic atypia, enlarged hyperchromatic nuclei, prominent nucleoli, and frequent mitotic figures, with tumor cells exhibiting mucin-rich cytoplasm. **(D)** Immunohistochemistry for Ki-67 showing an elevated proliferative index. **(E)** CDX2 demonstrating strong nuclear positivity in tumor cells. **(F)** CK20 showing diffuse cytoplasmic positivity. **(G)** HER2 expression status. **(H)** p53 demonstrating aberrant nuclear expression pattern. **(I)** Mucin staining highlighting abundant intracellular and extracellular mucin production.

Following a MDT discussion, chemotherapy with the FOLFOX regimen (oxaliplatin, leucovorin, and fluorouracil) was initiated on April 5, 2025. Concurrently, molecular profiling of the tumor was performed, identifying a rare *HER2* exon 17 c.G2033A p.R678Q mutation (36.52%) (**Table 1**). Given the established efficacy of trastuzumab in tumors harboring *HER2* mutations, trastuzumab was added to the treatment regimen on April 26, 2025. After four cycles of FOLFOX and trastuzumab therapy, follow-up contrast-enhanced CT imaging revealed tumor progression (**Figures 3A–C**). Following further MDT evaluation, the treatment regimen was altered to include oxaliplatin combined with albumin-bound paclitaxel. Despite two additional cycles of this new regimen, imaging confirmed continued disease progression (**Figures 3D–F**). Additionally, the patient developed severe anorexia, nausea, and vomiting due to both tumor progression and chemotherapy side effects. Faced with these debilitating symptoms and the lack of therapeutic benefit, the patient chose to discontinue further chemotherapy. Unfortunately, the patient passed away on December 27, 2025, as a result of continued disease progression.

**Table 1:** Alphabetical codes assigned to weed species, along with the number of labels and images for each class.

Gene	Functional region	Nucleotide Change	Amino Acid change	Variant Allele Frequency (VAF)
HER2	exon 17	c.G2033A	p.R678Q	36.52%
TP53	exon 4	c.121delG	p.D41Mfs*3	34.82%
ERBB4	exon 20	c.T2393G	p.L798R	9.37%
MYCL	–	–	Copy number gain	7 copies
KDM6A	exon 16	c.G1760A	p.S587N	19.63%
FGF14	exon 1	c.A106G	p.N36D	13.43%
FLT3	exon 8	c.A919G	p.R307G	12.44%
GABRA6	exon 9	c.T1271C	p.L424P	11.96%
YAP1	exon 4	c.T788C	p.L263P	10.63%
QKI	exon 7	c.A952G	p.K318E	9.82%
EPHA5	exon 3	c.A392C	p.K131T	9.38%
SF3B1	exon 19	c.C2831A	p.S944Y	8.10%
FGF2	exon 1	c.C205T	p.R69W	7.10%

VAF, Variant Allele Frequency; HER2, Human Epidermal Growth Factor Receptor 2; TP53, Tumor Protein p53; ERBB4, Erb-B2 Receptor Tyrosine Kinase 4; MYCL, MYCL Proto-Oncogene; bHLH, Transcription Factor; KDM6A, Lysine Demethylase 6A; FGF14, Fibroblast Growth Factor 14; FLT3, Fms Related Receptor Tyrosine Kinase 3; GABRA6, Gamma-Aminobutyric Acid Type A Receptor Subunit Alpha6; YAP1, Yes-Associated Protein 1; QKI, Quaking Homolog; KH, Domain RNA Binding; EPHA5, EPH Receptor A5; SF3B1, Splicing Factor 3b Subunit 1; FGF2, Fibroblast Growth Factor 2.

**Figure 3:**
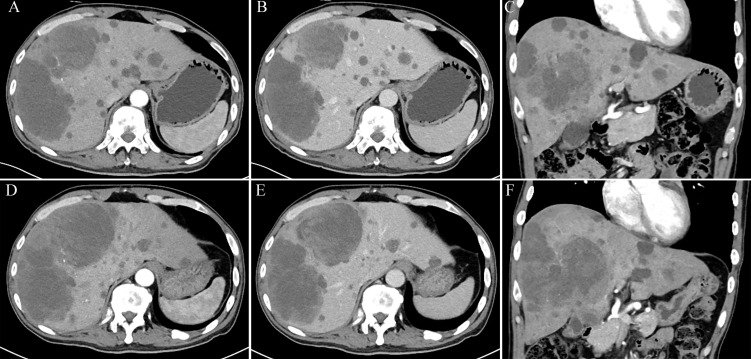
Radiological assessment following two sequential chemotherapy regimens. **(A–C)** Contrast-enhanced abdominal CT obtained after four cycles of the first-line chemotherapy regimen. **(D–F)** Contrast-enhanced abdominal CT obtained after two cycles of the second-line chemotherapy regimen. **(A)** Arterial-phase image demonstrating an increase in both the size and number of hepatic lesions compared with baseline imaging. **(B)** Portal venous-phase image confirming interval progression. **(C)** Coronal reconstructed image illustrating the extent of disease progression. **(D)** Arterial-phase image showing further enlargement and increased number of hepatic lesions compared with the pre–second-line baseline. **(E)** Portal venous-phase image. **(F)** Coronal reconstructed image demonstrating continued radiologic progression.

## Discussion

MAC most commonly originates in the colorectal region (9). However, in the present case, comprehensive diagnostic evaluations—including whole-body PET-CT and gastrointestinal endoscopy—failed to identify a primary lesion. This case represents a rare presentation of CUP with hepatic metastases, histologically confirmed as MAC with neuroendocrine differentiation and harboring an uncommon ERBB2 (HER2) R678Q mutation. To our knowledge, this is the first reported case of a HER2 R678Q-mutant MAC exhibiting neuroendocrine features without a detectable primary site. The case highlights critical diagnostic, molecular, and therapeutic challenges that warrant further exploration.

The differential diagnosis was challenging because MAC with neuroendocrine differentiation can originate from several primary sites. Metastatic colorectal MAC was the main consideration, given the elevated CEA level, multiple liver metastases, mucinous morphology, and CDX2/CK20 positivity. However, gastrointestinal endoscopy and biopsy did not identify a definite colorectal malignancy, and SATB2 negativity further weakened this diagnosis. Gastric and pancreaticobiliary origins were also considered, but gastric biopsy showed only mucosal inflammation, and imaging revealed no pancreaticobiliary primary lesion. Pulmonary invasive MAC and primary hepatic malignancy, including intrahepatic cholangiocarcinoma with mucinous features, were considered but were not supported by imaging or multidisciplinary evaluation. Although synaptophysin positivity raised the possibility of neuroendocrine carcinoma or mixed neuroendocrine–non-neuroendocrine neoplasm, the tumor was morphologically dominated by MAC, with negative CD56 and CgA expression. Therefore, the final diagnosis was metastatic MAC with neuroendocrine differentiation in the context of cancer of unknown primary.

The development of MACs is closely associated with the excessive accumulation of extracellular mucin (7, 8). Under physiological conditions, mucin—a complex glycoprotein—plays critical roles in inflammation and tissue repair (22). However, in malignancy, dysregulated mucin expression facilitates immune evasion and disrupts normal cell–cell adhesion (23). In particular, aberrant expression, glycosylation, and mislocalization of transmembrane mucins—such as MUC1, MUC4, and MUC16—can interfere with cell signaling, promote cancer cell proliferation, inhibit apoptosis, and drive metastasis through interactions with growth factor receptors and immune-modulatory pathways (24). These molecular alterations transform mucins from passive epithelial barriers into active mediators of tumorigenesis and cancer progression. By reshaping the tumor microenvironment, they support anchorage-independent growth, enhance cellular plasticity, and ultimately promote tumor invasion and metastatic spread (24, 25). MACs are characterized by distinct molecular landscapes that vary according to the tissue of origin. In pulmonary MACs, common genomic features include a high frequency of *KRAS* mutations, frequent *TP53* alterations, and a relative paucity of *APC* mutations compared with non-mucinous colorectal carcinomas, along with low levels of chromosomal instability and frequent associations with microsatellite instability (MSI) and the CpG island methylator phenotype (10). In contrast, intestinal MACs demonstrate a higher prevalence of *KRAS* mutations and a lower tendency toward *BRAF* mutations (9). Notably, patients with MAC exhibit an increased rate of MSI-high status, a molecular characteristic that may provide a biological rationale for the use of immunotherapy in this subtype (9, 26).

MACs are frequently diagnosed at an advanced stage and are significantly associated with higher TNM classifications and an increased likelihood of lymph node metastasis (9, 27, 28). These clinical features reflect the aggressive nature of MAC and underscore the pivotal role of mucin in facilitating local tumor invasion and metastatic spread. In colorectal cancer, the prognostic implications of MAC vary markedly depending on tumor location (27). While colonic MACs generally exhibit overall survival rates comparable to those of non-mucinous adenocarcinomas, rectal MACs are consistently linked to significantly worse outcomes (27, 28). This disparity may, in part, be attributed to distinct immunophenotypic profiles. Colorectal MACs, particularly those arising in the rectum, are characterized by high expression of MUC2 and Bcl-2, alongside low nuclear p53 staining—molecular features associated with more aggressive tumor behavior (29, 30). Importantly, even after adjusting for stage and age, rectal MACs demonstrate a significantly reduced cancer-specific survival compared to their non-mucinous counterparts (27, 29). Tumor location thus emerges as a critical prognostic factor in colorectal MACs, with rectal lesions carrying a 1.3- to 2-fold higher risk of cancer-specific mortality (29, 31). In contrast, MACs located in the right colon are often associated with equivalent or slightly improved survival outcomes relative to conventional adenocarcinomas, suggesting that site-specific molecular biology exerts a substantial influence on disease progression and therapeutic responsiveness (27, 29). Moreover, MACs have been shown to exhibit reduced sensitivity to both chemotherapy and chemoradiotherapy (CRT) compared with non-mucinous adenocarcinomas (NMACs) (32). This diminished responsiveness is thought to result from several factors, including lower proliferative activity, abundant extracellular mucin that may hinder drug and radiation penetration, and distinct molecular features that contribute to treatment resistance (32). Collectively, these characteristics may underlie the poorer prognosis typically observed in patients with MAC.

HER2 (ERBB2) is a member of the EGFR/ERBB family of receptor tyrosine kinases and plays a critical role in regulating cell growth, differentiation, and survival (33). Unlike other ERBB family members, HER2 lacks a known ligand and adopts a constitutively active, untethered extracellular domain (ECD) conformation (34, 35). This structural feature enables HER2 to readily form heterodimers—particularly with ligand-bound, untethered EGFR or HER3—which potently activates downstream signaling pathways (35). Amplification and overexpression of ERBB2 act as oncogenic drivers in approximately 20% of human breast and gastric cancers. Given its well-established role as a potent oncogene, HER2 has become a major therapeutic target (36). To date, several HER2-targeted agents have been approved by the U.S. Food and Drug Administration (FDA), including three monoclonal antibodies—trastuzumab, pertuzumab, and the antibody–drug conjugate ado-trastuzumab emtansine (T-DM1)—as well as two small-molecule tyrosine kinase inhibitors, lapatinib and neratinib, for the treatment of HER2-driven malignancies (37). In the present case, a rare HER2 R678Q mutation was identified in tumor samples from a patient with MAC, suggesting a potential role for this alteration in tumorigenesis and therapeutic response. The HER2 R678Q mutation, located within the juxtamembrane domain of the receptor, has previously been characterized as a potentially activating alteration that enhances HER2-driven signaling, promotes anchorage-independent growth, and supports tumor formation *in vivo (*37, 38). Beyond its oncogenic effects, this mutation has also been associated with a significantly reduced response to oxaliplatin-based chemotherapy in patients with gastric cancer, likely due to mutation-induced conformational changes in the receptor that alter downstream signaling pathways involved in DNA damage repair or drug detoxification (38). Consistent with prior observations, our patient similarly demonstrated early resistance to oxaliplatin-based chemotherapy, with disease progression observed shortly after treatment initiation. Previous studies have reported that the HER2 R678Q mutation does not significantly influence responses to ramucirumab or immune checkpoint inhibitors such as nivolumab, suggesting that this alteration confers a mutation-specific pattern of chemoresistance rather than reflecting a globally aggressive tumor phenotype (38). In our case, immunotherapy was not administered; however, despite the addition of trastuzumab to systemic chemotherapy, no meaningful clinical benefit was observed. These findings further support the notion that the HER2 R678Q mutation may be associated with limited sensitivity to both platinum-based chemotherapy and HER2-targeted monoclonal antibody therapy in MAC, underscoring the need for alternative therapeutic strategies.

This case report presents a unique instance of MAC with neuroendocrine differentiation and a rare HER2 R678Q mutation, but several limitations must be acknowledged. First, as a single-patient case study, the generalizability of the findings is inherently limited. The clinical behavior, therapeutic response, and molecular characteristics observed may not be representative of broader patient populations with similar pathologies. Second, due to the unknown primary origin, complete clinicopathological correlation and accurate tissue-of-origin diagnosis could not be established, which may influence interpretation of treatment response and prognosis. Although comprehensive diagnostic workup was performed, including advanced imaging, endoscopic evaluation, and molecular profiling, the absence of a confirmed primary site constrained the formulation of site-specific therapeutic strategies. Third, while the identification of the HER2 R678Q mutation provides valuable insight, functional validation of this mutation’s role in tumorigenesis and chemoresistance was not conducted *in vitro* or *in vivo*. As a result, conclusions regarding its clinical significance remain speculative and hypothesis-generating. Additionally, despite the incorporation of trastuzumab into the treatment regimen, therapeutic resistance was observed, suggesting a need for further exploration of HER2-targeted approaches specific to rare mutations.

## Conclusion

This case highlights a rare presentation of MAC with neuroendocrine differentiation and a novel HER2 R678Q mutation in the context of CUP. The identification of the HER2 R678Q mutation offers new insight into the molecular landscape of mucinous neoplasms and raises important questions regarding mutation-specific therapeutic resistance. Although trastuzumab was administered based on HER2 mutation status, the lack of clinical response emphasizes the need for further research into effective targeted therapies for rare HER2 variants.

## Data Availability

The original contributions presented in the study are included in the article/supplementary material. Further inquiries can be directed to the corresponding author.
